# Optimization of Zika virus envelope protein production for ELISA and correlation of antibody titers with virus neutralization in Mexican patients from an arbovirus endemic region

**DOI:** 10.1186/s12985-018-1104-6

**Published:** 2018-12-27

**Authors:** Young Chan Kim, Cesar Lopez-Camacho, Joanne E. Nettleship, Nahid Rahman, Michelle L. Hill, Laura Silva-Reyes, Georgina Ortiz-Martinez, Gloria Figueroa-Aguilar, María Antonieta Mar, Héctor Vivanco-Cid, Christine S. Rollier, Nicole Zitzmann, Martha Eva Viveros-Sandoval, Raymond J. Owens, Arturo Reyes-Sandoval

**Affiliations:** 1 The Jenner Institute, Nuffield Department of Medicine, The Henry Wellcome Building for Molecular Physiology, University of Oxford, Old Road Campus Research Building. Roosevelt Drive, Oxford, OX3 7DQ UK; 2 Division of Structural Biology, Wellcome Centre for Human Genetics, University of Oxford, Roosevelt Drive, Oxford, UK; 3 Rutherford Appleton Laboratory, OPPF-UK, Research Complex at Harwell, Oxford, UK; 4 Oxford Vaccine Group, Department of Paediatrics, University of Oxford and the NIHR Oxford Biomedical Research Centre, Centre for Clinical Vaccinology and Tropical Medicine, Churchill Hospital, Oxford, UK; 5 Laboratorio de Hemostasia y Biología Vascular. División de Estudios de Posgrado. Facultad de Ciencias Médicas y Biológicas “Dr. Ignacio Chávez”, Universidad Michoacana de San Nicolás de Hidalgo, UMSNH, Morelia, Mexico; 6 UMSNH–Oxford University of Oxford Clinical Research Laboratory (UMOCRL), Faculty of Biological and Medical Sciences “Dr. Ignacio Chávez”, Universidad Michoacana de San Nicolás de Hidalgo, Morelia, Michoacán Mexico; 7 Oxford Glycobiology Institute, Department of Biochemistry, University of Oxford, South Parks Road, Oxford, OX1 3QU UK; 8 Laboratorio Estatal de Salud Pública, Secretaría de Salud de Michoacán, Morelia, Michoacán Mexico; 9 Instituto de Investigaciones Médico-Biológicas, Universidad Veracruzana, Veracruz, Mexico; 10 HGZMF No. 12 Lázaro Cárdenas Michoacán dirección av. Lázaro Cárdenas No. 154 Col. Centro Lázaro Cárdenas Michoacán, Veracruz, Mexico

**Keywords:** Zika virus, Envelope protein, CD4 fusion tag, Protein production, ELISA, Mexican patients, Neutralizing antibodies

## Abstract

**Background:**

Zika virus (ZIKV) has become a global threat with immediate need for accurate diagnostics, efficacious vaccines and therapeutics. Several ZIKV envelope (Env)-based vaccines have been developed recently. However, many commercially available ZIKV Env are based on the African lineage and produced in insect cells. Here, we sought to produce Asian-lineage ZIKV Env in mammalian cells for research and clinical applications.

**Methods:**

We designed various gene expression constructs to optimize the production of ZIKV using prM-Env and full or C-terminal truncations of Env; with or without a rat CD4 fusion partner to allow large-scale production of soluble protein in mammalian HEK293 cells. Protein expression was verified by mass spectrometry and western-blot with a pan-flavivirus antibody, a ZIKV Env monoclonal antibody and with immune sera from adenoviral (ChAdOx1) ZIKV Env-vaccinated mice. The resulting Env-CD4 was used as a coating reagent for immunoassay (ELISA) using both mouse and human seropositive sera.

**Results:**

Replacement of the C-terminus transmembrane Env domain by a rat CD4 and addition of prM supported optimal expression and secretion of Env. Binding between the antigens and the antibodies was similar to binding when using commercially available ZIKV Env reagents. Furthermore, antibodies from ZIKV patients bound ZIKV Env-CD4 in ELISA assays, whereas sera from healthy blood donors yielded minimal OD background. The serological outcomes of this assay correlated also with ZIKV neutralisation capacity in vitro.

**Conclusions:**

Results obtained from this study indicate the potential of the Asian-lineage Zika Env-CD4 and Env proteins in ELISA assays to monitor humoral immune responses in upcoming clinical trials as well as a sero-diagnostic tool in ZIKV infection.

**Electronic supplementary material:**

The online version of this article (10.1186/s12985-018-1104-6) contains supplementary material, which is available to authorized users.

## Background

Zika virus (ZIKV) is an emerging arthropod-borne virus that belongs to the family *Flaviviridae* and the genus Flavivirus. It was first discovered in a sentinel rhesus monkey in the Zika forest of Uganda in 1947 [1]. ZIKV is classified into two lineages: African and Asian strains [2] but they share > 95% amino acid identity with a single serotype unlike the closely related flavivirus, dengue virus (DENV) that is composed of 4 different serotypes [3]. Since its first detection in Brazil in 2015 [4], ZIKV has spread rapidly throughout the Americas, and over 170,000 laboratory cases had been confirmed in 48 countries worldwide by the end of 2016 [5]. ZIKV is mainly transmitted via mosquitoes of the genus *Aedes* [6]***,*** but also through sexual contact [7] and vertical transmission [8, 9]. Most of ZIKV infections are asymptomatic or mild [10], but in some cases can lead to neurological complications including Guillain-Barré syndrome (GBS) in adults and microcephaly in foetuses [8, 9, 11].

ZIKV is an enveloped virus with a ~ 10.7 Kb single-stranded, positive-sense RNA genome which encodes a single viral polyprotein that is processed post-translationally into three structural proteins (capsid [C], pre-membrane [prM] and envelope [Env]) and seven non-structural proteins (NS1, NS2A, NS2B, NS3, NS4A, NS4B and NS5) [12, 13]. Recent cryo-electron microscopy analysis of ZIKV revealed that the overall structure is nearly identical to other closely related flaviviruses such as dengue (DENV) [14, 15]. Env is involved in receptor binding, fusion and viral entry into target cells and is a primary target for neutralizing antibodies during ZIKV infection [16, 17]. prM interacts with the nascent Env protein during the viral assembly to assist correct folding and prevent premature fusion to the endoplasmic reticulum membrane. It does this by covering the fusion loop in Env before eventually releasing the prM to form mature virions [18]. The expression and purification of soluble recombinant Asian-lineage ZIKV Env proteins have been described using *E. coli* as the host cell and involve expression as inclusion bodies and re-folding in vitro [19]. Expression using *Drosophilia* cells has also been described [20]. In addition, expression of African ZIKV Env proteins in mammalian cells such as HEK293T cells has been described more recently and the N-glycosylation on ZIKV Env was found to be important for expression and secretion of Env [21–23]. Many commercially available ZIKV Env proteins are baculovirus-derived and expressed in insect cells based on sequences from the African strain, with a purity of > 85% for ELISA applications, but they may not be easily affordable in Zika endemic areas in developing countries. Protein expression in a mammalian system is more challenging than in *E. coli* or in insect cells due to lower yield and increased cost. Use of a mammalian platform however, ensures correct post-translational modifications [24, 25] including glycosylation at N154 of ZIKV Env which is an important determinant of ZIKV virulence and neuro-invasive potential [26]. Previous studies have investigated the use of fusion partners such as maltose binding protein (MBP) fused to dengue EDIII or rat CD4 fused to the CD5 immunoglobulin domain to enhance protein solubility and secretion but there is no data available that describes the use of fusion partners for Zika Env [27–29].

We have recently developed adenovirus based (ChAdOx1 ZIKV) vaccines, which induce strong immune response in mice and provide protection in a mice ZIKV challenge model [30]. An Asian-lineage Env antigen would be ideal for ELISA assays to monitor the humoral immune response in ZIKV patients and in human volunteers in upcoming clinical trials; as well as to monitor responses in ZIKV pre-clinical models including mice and non-human primates. Such assays could also have a potential use for sero-diagnosis of Zika infection, as these strains of Asian-American lineage are currently causing outbreaks in the Americas.

In this study, we have designed various constructs (prM-Env, Env only and truncations of Env) with or without a rat CD4 fusion tag at the C-terminus to optimise large-scale production of soluble recombinant Asian-lineage ZIKV Env proteins. Production of Env is intended for both Zika disease diagnosis and monitoring of humoral responses in both mice and humans vaccinated with Env-based vaccines. The correct expression of Env-CD4 and Env proteins was verified by western blot using a mouse pan-flavivirus monoclonal antibody, a ZIKV Env monoclonal antibody, and by ELISA assays using sera from mice immunized with a ChAdOx1 ZIKV vaccine. The recombinant Env-CD4 was able to bind antibodies from patient sera with Zika infection and distinguished from human sera without previous exposure to flaviviral infections. This serological data was also correlated with ZIKV neutralisation capacity in vitro.

## Results

### CD4 fusion tag increases the secretion of ZIKV Env proteins in mammalian cells

**Fig. 1:**
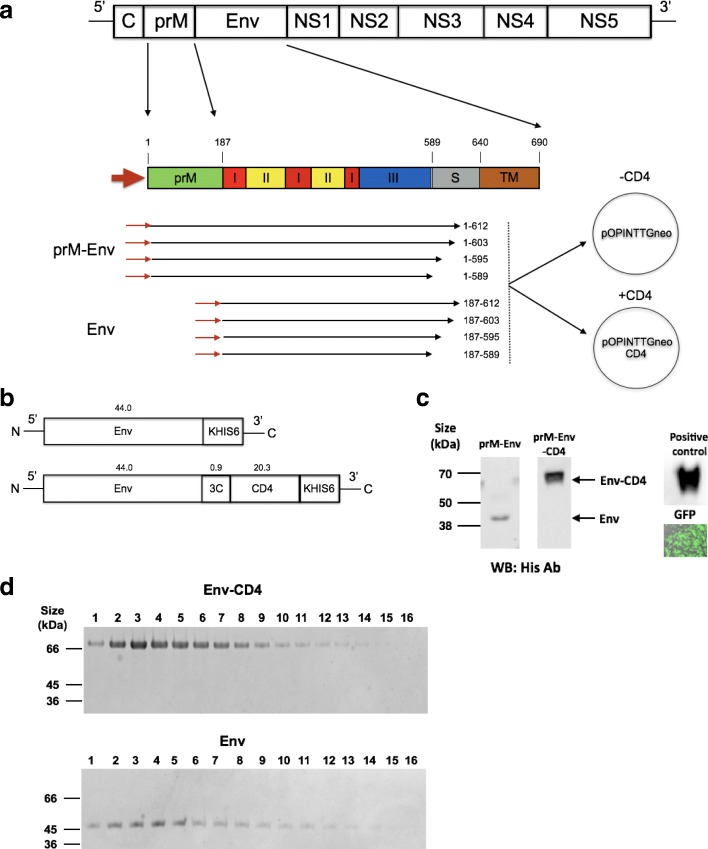
Design and production of Zika Envelope proteins. **a**. Design of ZIKV envelope constructs. The full genome of ZIKV is shown. The genes for pre-membrane (prM) and envelope (Env) including the 3 domains DI, DII, DIII, stem (S) and transmembrane regions (TM) are highlighted. The red arrow indicates the direction of transcription. 8 plasmid constructs encoding prM-Env or Env were designed and cloned into either pOPINTTGneo or pOPINTTGneo-3C-CD4 expression vectors resulting in total of 16 constructs. **b**. The structure of secreted Env proteins: (top) prM-Env (1–589) in pOPINTTGneo produces Env protein with a His-tag after cleavage of prM upon secretion. (bottom) prM-Env (1–589) in pOPINTTGneo-3C-CD4 produces Env protein with 3C protease site, CD4 fusion tag and His tag. The approximate sizes of components of Env and Env-CD4 proteins are shown in kDa. **c**. An example of western-blot of Env-CD4 (62.5 kDa) and Env (44 kDa) secreted from HEK293T and detected with anti-His antibodies for constructs encoding prM-Env and prM-Env-CD4 are shown. The positive control was the ectodomain of cell surface receptor sFcεRIα [46] and the negative control was GFP. **d**. Coomassie Brilliant Blue staining (CBB) of the fractions of purified Env-CD4 and Env proteins are shown

We designed several expression plasmids for production of soluble Asian-lineage ZIKV Env (Fig. 1). Constructs encoded prM-Env or Env with different C-terminal truncations within the stem region of ZIKV envelope which extended from residue 589 into the membrane linker or STEM region, thus ablating the expression of the transmembrane domain (TM) (Fig. 1a). Residue 589 was chosen by structural alignment of the ZIKV Env sequence of an isolate from the 2013 French Polynesian ZIKV outbreak with that of DENV (Additional file 1) [31]. These constructs were cloned either into pOPINTTGneo or pOPINTTGneo-3C-CD4 expression vectors to produce soluble ZIKV Env proteins with a C-terminal His tag or a C-terminal rat CD4 fusion and His tag, respectively (Fig. 1a, b). A 3C protease site was added in Env-CD4 protein to allow cleavage of the CD4 molecule following purification. A series of small-scale expression tests were carried out in HEK293T cells and the secreted Env proteins were analysed by western-blot using an anti-His tag antibody (Fig. 1c). The Env protein secretion profiles from three small-scale expressions are summarised in Additional file 2, and indicate that the expression and secretion of ZIKV Env proteins were improved by the addition of the rat CD4 tag and also by the presence of prM. The constructs encoding ZIKV prM-Env (amino acid residues 1–589) showed the best secretion profiles in small scale HEK293T cells, and therefore were selected to be expressed at larger scale using the Expi293 mammalian transient expression system (ThermoFisher Scientific). After purification by nickel column chromatography and size exclusion chromatography, the purified fractions containing ZIKV envelope proteins (Env-CD4 and Env) were analysed by SDS-PAGE with Coomassie Brilliant Blue (CBB) staining (Fig. 1d) and mass spectrometry (data not shown). Interestingly, the total yield of ZIKV Env-CD4 protein (~ 3.3 mg/L) was > 5–10 folds higher than the yield of Env protein (~ 0.3–0.66 mg/L) from multiple separate Expi293 expressions which indicate that the use of the CD4 fusion tag is an effective strategy to increase the secreted expression of ZIKV envelope proteins in mammalian cells.

### Characterisation of recombinant Asian-lineage ZIKV Env-CD4 and Env proteins

**Fig. 2:**
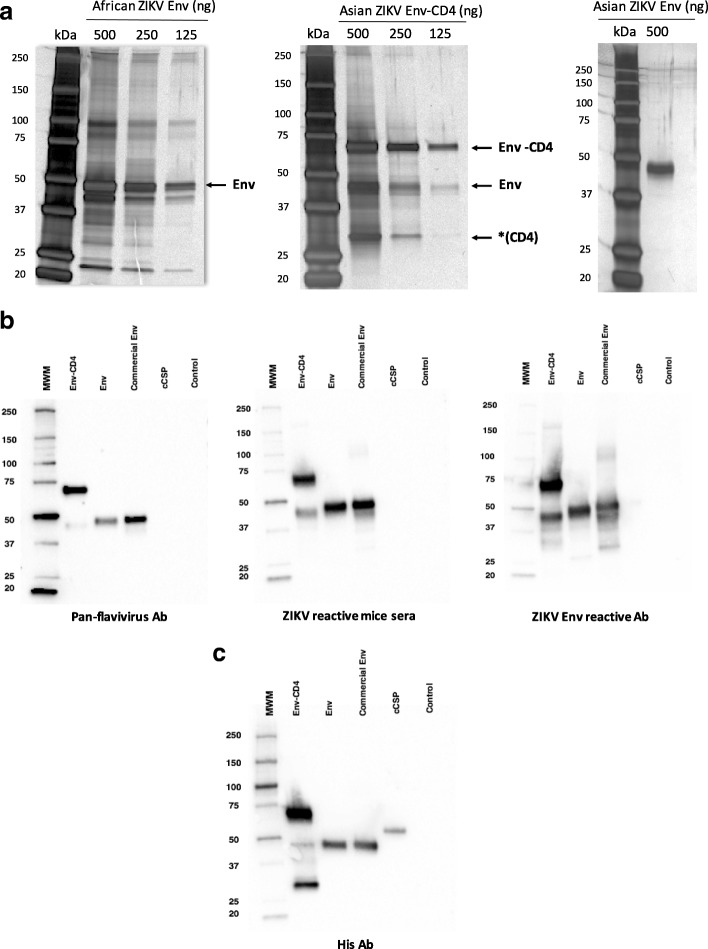
Characterisation of ZIKV Env proteins. **a**. Silver stained PAGE gel showing purity of ZIKV Env proteins: commercial Env based on African Strain (left panel), Env-CD4 (middle) and Env (right) based on Asian-lineage strain. Three concentrations of Env proteins were used: 500 ng, 250 ng and 125 ng for African ZIKV Env (commercial) and Asian-lineage ZIKV Env-CD4. Env-CD4 showed three distinctive bands at ~ 65 kDa, ~ 45 kDa and ~ 25 kDa on the gel, corresponding to Env-CD4, Env and CD4 fusion tag (CD4), respectively. CD4 fragment is liberated following cleavage from Env-CD4 protein into Env and CD4. **b**. Western blot of the purified ZIKV Env-CD4 and Env proteins using a pan-flavivirus antibody, sera from ChAdOx1 ZIKV prME_ΔTM immunized mice and Zika Env monoclonal antibody. For Env-CD4, the top band corresponds to Env-CD4 protein and the bottom band to Env protein which may be formed following cleavage from Env-CD4. **c**. Western blot of the purified Env-CD4 using an anti-His antibody (1:2000). For Env-CD4, the top band is Env-CD4 protein and bottom band corresponds to CD4 (His). An unrelated malaria protein (cCSP-His) was used as positive control for the His antibody and as a negative control for the ZIKV-Env specific antibodies. Negative control is cell-free media

PAGE gels were stained with silver stain to assess the purity of the recombinant Asian-lineage ZIKV Env-CD4 and Env proteins, and to compare with a commercially available ZIKV Env protein (African strain) (Fig. 2a). The commercial ZIKV Env protein showed a band at approximately 45 kDa, which corresponds to Env protein with high-molecular bands that could represent homodimers that resisted reducing conditions (Fig. 2a, left) and some lower size bands. In contrast, our purified ZIKV Env showed a single band at ~ 45 kDa on a silver stained PAGE gel which indicated higher purity of our ZIKV Env protein when compared to that of the commercially available reference sample (Fig. 2a, right). Env-CD4 was found to contain three distinctive bands at ~ 65 kDa, ~ 45 kDa and ~ 25 kDa on the gel, corresponding to Env-CD4, Env and CD4 fusion tag, respectively (Fig. 2a, middle). This suggests that some CD4 fusion tag may undergo cleavage from Env-CD4 protein into Env and CD4, possibly due to cross-reactive protease enzymes in the cells and under reducing conditions. To test that there is less degree of CD4 fusion tag cleavage into Env and CD4 at non-reducing conditions, a PAGE-silver stain was carried out which detected a single band ~ 65 kDa (Env-CD4) at 250 ng at non-reducing condition (Additional file 3). We verified the Asian-lineage ZIKV Env-CD4 recombinant protein by western blotting using a pan flavivirus antibody, the sera from ChAdOx1 ZIKV prME_ΔTM vaccinated mice and a ZIKV Env monoclonal antibody (Fig. 2b). Two bands around 65 kDa and 45 kDa were detected which correspond to Env-CD4 and Env protein lacking the CD4 fusion tag, respectively. All purified ZIKV Env proteins characterised by western blotting recognized a ~ 45 kDa single band by three different antibodies. To confirm the identity of the protein band at ~ 25 kDa (Fig. 2a, middle) as the CD4 cleavage product, a western blot was carried out using a His-tag antibody which detected two bands of ~ 65 kDa (Env-CD4) and ~ 25 kDa (CD4-His) both of which would be expected to have His tag (Fig. 2c). There was a faint band ~ 45 kDa which would be expected size of Env and this is likely to be due to small amount of Env crossing-over from the adjacent well on the gel. This was confirmed by repeating western blot of Env-CD4 using a His-tag antibody which showed two bands ~ 65 kDa (Env-CD4) and ~ 25 kDa (CD4-His) but no band ~ 45 kDa (data not shown).

### Assessing the reactivity of ZIKV Env-CD4 and Env by ELISA

**Fig. 3:**
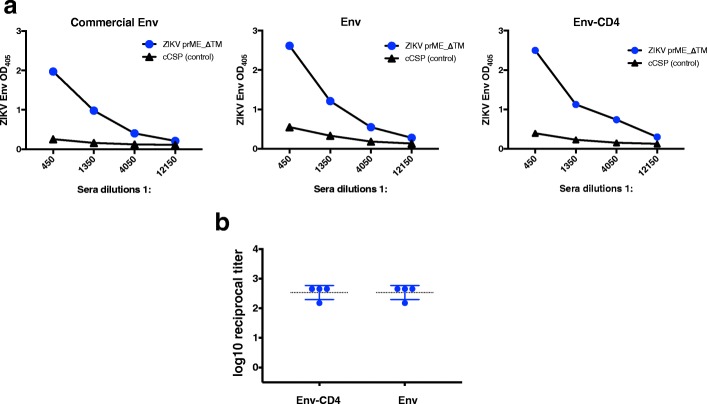
Immunological ELISA assays against sera from adenoviral vaccinated mice. **a**. ELISA assays of commercial African-lineage ZIKV Env vs Asian-lineage ZIKV Env and Env-CD4. Humoral responses in pool of mice sera (*n* = 6) vaccinated with a ChAdOx1 ZIKV prME_ΔTM vaccine and the control which is the unrelated antigen (cCSP). **b**. ELISA assays of endpoint reciprocal titer to measure antibodies in mice sera (*n* = 4) vaccinated with prME_ΔTM by Env-CD4 or Env proteins. Individual data are shown (blue circles), and mean + SD are represented as the horizontal bars

Next, we sought to explore whether ZIKV Env-CD4 and Env proteins could bind to antibodies elicited upon ChAdOx1 ZIKV vaccination in mice, and to compare this result to that of the commercially available ZIKV Env protein, in an ELISA assay. We compared commercial ZIKV Env protein and ZIKV Env-CD4 recombinant protein by ELISA against a pool of ZIKV mouse immune sera obtained 3-months post immunization with a ChAdOx1 ZIKV prME_ΔTM based on Asian-lineage ZIKV strain, and using an unrelated ChAdOx1 vaccine as a control (malarial cCSP) (Fig. 3a). In our recent study assessing the immunogenicity of four replication-deficient chimpanzee adenoviral (ChAdOx1) ZIKV vaccine candidates, ChAdOx1 prME_ΔTM vaccine induced the highest antibody titers by ELISA assay using a commercial ZIKV ELISA kit [30]. In this study, the ELISA assay using recombinant Env-CD4 protein also detected antibodies from mouse sera after ZIKV (prME_ΔTM) vaccination, with similar or higher ODs to that of the commercially available ZIKV Env (Fig. 3a). In addition, quantification of antibody binding to ZIKV Env protein and Env-CD4 protein using sera from mice (*n* = 4) after vaccination with prME_ΔTM at greater dilution range (1:50–1:12150) yielded a similar mean reciprocal titer (Fig. 3b). These results indicate that both Env and Env-CD4 proteins are recognised by antibodies raised after vaccination with prME_ΔTM and the presence of CD4 fusion tag does not impair binding of ZIKV-specific antibodies. We conclude that purified ZIKV Env-CD4 and Env recombinant proteins are suitable for immunological assays to assess humoral responses in mice.

### ZIKV antibodies from patients that had a confirmed ZIKV infection are able to bind ZIKV Env-CD4

**Fig. 4:**
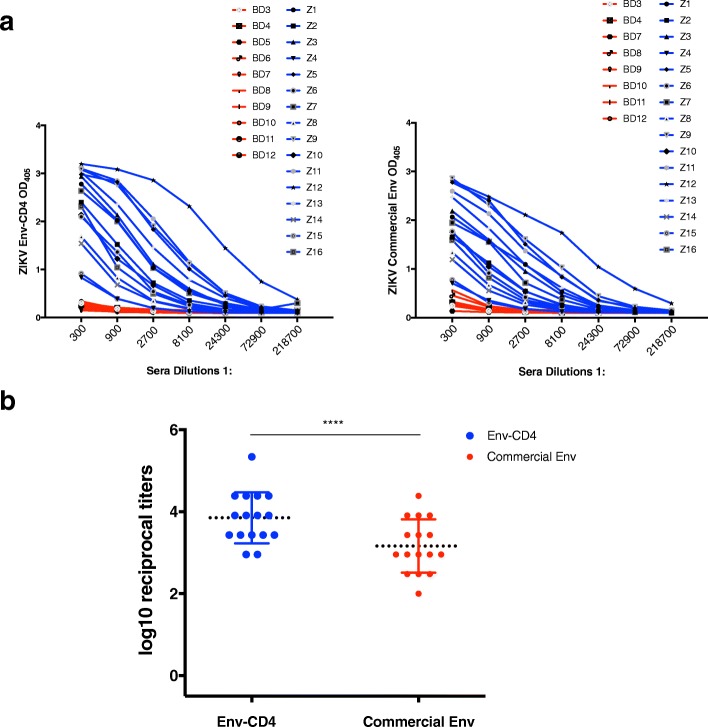
ELISA assays to assess the reactivity of human sera from patients in a ZIKV endemic region of Mexico. Sera from Zika patients from Mexico (blue) and sera obtained from healthy donors (who should have less or no previous flaviral exposure (red). **a**. The graph of ZIKV Env-CD4 vs commercial Env OD405 against sera dilutions. **b**. The graph shows the endpoint reciprocal titers for ZIKV patients using Env-CD4 and commercial Env proteins. *P* value (< 0.0001) was determined by pairwise t-tests

ELISA assay using sera from ZIKV-infected patients and from an endemic area was used to test whether our Asian-lineage ZIKV Env-CD4 protein could be used to detect patient-derived ZIKV antibodies. We collected sera from 16 patients who had ZIKV infection confirmed by real-time RT-PCR and sera from ten healthy blood donors were tested in parallel (Fig. 4a). The ODs demonstrate that both Env-CD4 and commercial Env can reliably detect and differentiate ZIKV-infected antisera from control sera (Fig. 4a). Sera from Z12 had the highest OD and thus potentially with high levels of ZIKV Env antibodies. Endpoint reciprocal titers (Fig. 4b) showed that all patients with previous ZIKV infection have high mean titer of 3.84 and Z12 had the highest titer of 5.34 using Env-CD4 whilst commercial Env showed a significantly lower mean titer of 3.16 and Z12 had the titer of 4.38. These results indicate that our purified Asian-lineage ZIKV Env-CD4 recombinant protein can reliably detect anti-ZIKV Env antibodies in infected individuals, which may be relevant for sero-diagnosis of ZIKV infection, as well as its application in the monitoring of vaccine immunogenicity, during ZIKV vaccine clinical trials in healthy volunteers.

### ZIKV antibodies from previously ZIKV-infected patients correlated to the level of neutralizing antibodies

**Fig. 5:**
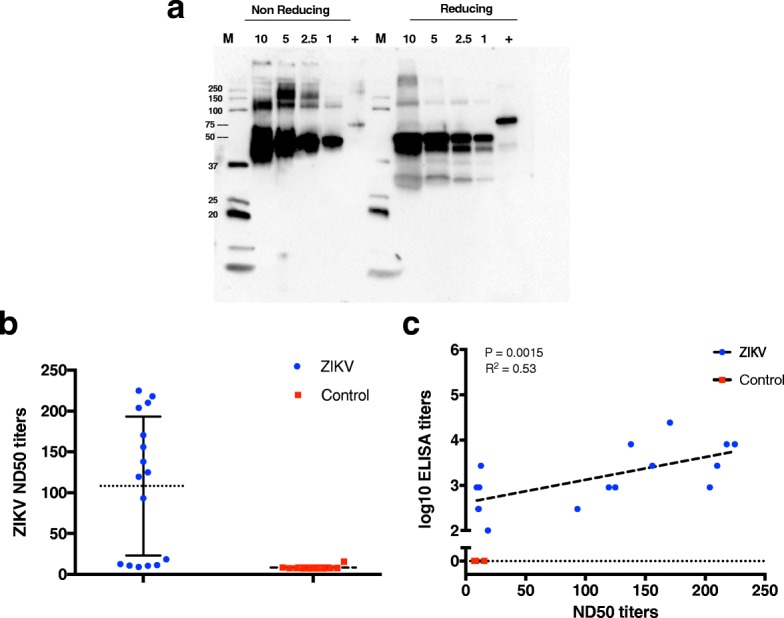
ZIKV PRNT assays assessing the level of neutralising antibodies in human sera from ZIKV infected patients. **a**. ZIKV preparation for the neutralization assay was subjected to western-blot under reducing and non-reducing conditions. Different volumes (ul) were loaded in decreasing volumes and bands detected by an anti-ZIKV specific antibody. Positive control is ZIKV Env-CD4. **b**. ZIKV ND50 titers for sera from ZIKV infected patients and control sera. Sera from Zika patients (blue) and sera obtained from healthy donors (who should have less or no previous flaviviral exposure) (red) **c**. Correlation between the endpoint reciprocal titers from the ELISA assay and ND50 titers from the PRNT assay for ZIKV patients compared to the healthy donors. *P* values and R^2^ values reflect Pearson correlation tests

Sixteen sera samples from the ZIKV-infected patients and ten control sera were tested in duplicate (1:10 to 1:160, using 2-fold dilutions) by a ZIKV plaque reduction and neutralisation test (PRNT). To this end, Polyethylene glycol (PEG)-precipitated ZIKV was subjected to western-blot analysis (Fig. 5a) using a ZIKV Env monoclonal antibody as probe. Several higher molecular bands were detected, in agreement with the reduced and non-reduced conditions of the sample, thus confirming that the virus used for neutralisation is actually ZIKV. Then we assessed ZIKV antibody neutralisation activity in vitro and correlated the endpoint reciprocal antibody titer with the level of neutralising antibodies. The average ND50 titer for ZIKV-infected patient sera was 108, with 94% of samples showing an ND50 > 10. Nine controls showed ND50 < 10, with one (BD4) showing an ND50 titer of 15.5 (Fig. 5b). High levels of neutralising antibodies with ND50 > 100 were seen in sera from nine ZIKV-infected patients (Z12, Z5, Z11, Z13, Z7, Z3, Z2, Z7 and Z16). This correlated with ELISA reciprocal titer values by the Pearson Correlation Test (R^2^ = 0.53 and *P* value = 0.0015) as shown in Table 1 and Fig. 5c. Z5, Z11 and Z13 showed ND50 > 200 (Table 1), with endpoint reciprocal titers of 4.38 by ELISA. Z12 was found to have the highest endpoint titer of 5.34 by ELISA and also showed a high ND50 titer of 170.6. Three sera samples (75%) with endpoint reciprocal titers of 3.9 provided ND50 > 100, while only 2 out of 4 sera with titers of 3.43 demonstrated some evidence of neutralising antibodies, albeit at lower ND50s. These data suggest that high reciprocal titers from the ELISA assay correlate with neutralising titers (ND50) and a reciprocal endpoint titer > 4.38 corresponds to a higher level of neutralising antibodies in patient sera compared to samples with lower reciprocal titers. This may be relevant during ZIKV vaccine clinical trials to monitor the levels of neutralising antibodies against ZIKV Env upon vaccination. Similar correlation between ELISA titers using commercial ZIKV Env and ND50 titer also demonstrated a positive correlation but lower R^2^ (0.43) and *P* value (0.0057) (data not shown).

**Table 1:** List of all human sera descriptions and their respective ELISA endpoint reciprocal titers and ND50 titers

Human sera description	Zika patient number	ELISA log_10_ reciprocal titer	ND50 titer
Amb45	Z12	5.34	170.6
Amb24	Z5	4.38	224.8
Amb40	Z11	4.38	218
Amb74	Z13	4.38	210.1
Z7	Z7	3.9	203.9
BD32	Z3	3.9	155.9
Z2	Z2	3.9	119.5
Z1	Z1	3.9	12.7
Amb39	Z7	3.6	138
Amb92	Z14	3.43	93.3
Z8	Z8	3.43	10.8
Z10	Z10	3.43	< 10
Amb21	Z4	2.95	18.5
Amb130	Z15	2.95	10.5
Amb155	Z16	2.65	124.9
Amb33	Z6	2.65	11.3
BD3, BD5–12	N/A	0	< 10
BD4	N/A	0	15.5

## Discussion

Given the increasing importance of accurate sero-diagnosis of ZIKV infection and the necessity of monitoring humoral immune responses following vaccination with ZIKV vaccines in preclinical and clinical development, we have produced an Asian-lineage ZIKV Env recombinant protein with an optimized secretory profile in mammalian cells, to facilitate large-scale production. The rat CD4 fusion tag fused at the Env protein C-terminus increased secretion of Env-CD4, in-keeping with data for immunoglobulin CD48 [29] and the addition of prM also enhanced its secretion [32, 33]. The silver stained PAGE- profiles of purified ZIKV Env-CD4 and Env proteins indicates improved purity when compared with the commercially available African ZIKV Env protein produced in an insect system. Purified Env-CD4 and Env proteins were shown to bind to a pan-flavivirus antibody, to serum antibodies from mice immunized with adenovirus vectored ChAdOx1 ZIKV prME_ΔTM vaccines [30], and to a ZIKV Env monoclonal antibody, therefore confirming that the purified proteins are correctly expressed. A portion of ZIKV Env-CD4 was found to undergo auto-cleavage of CD4 and release of ZIKV Env and CD4 peptides under reducing conditions, but this was limited under the non-reducing conditions, which suggests that Env-CD4 may retain its intact structure under non-reducing conditions including in ELISA assays. Our novel ZIKV Env-CD4 and Env proteins are based on the Asian-lineage strain which is currently circulating in the Americas [4, 34, 35]. ELISA assays with these proteins using sera from mice immunized with adenovirus vectored ChAdOx1 ZIKV prME_ΔTM indicated that antibodies elicited by this vaccine are capable of binding Env-CD4 and Env, to a similar extent as binding to a commercial African lineage ZIKV Env.

The 3C protease site in Env-CD4 allows cleavage of CD4 following secretion and purification, however the presence of the CD4 fusion tag had no effect on the binding of ZIKV Env specific antibodies as shown by WB and ELISA. This suggests we can produce either ZIKV Env-CD4 or Env recombinant proteins for future use in sero-diagnosis of ZIKV and in future clinical trials or in further vaccine efficacy assessments. The ELISA results demonstrated that Env-CD4 was recognised by antibodies in ZIKV-exposed patients and with higher ODs than sera from healthy donors. The mean endpoint reciprocal titers for Env-CD4 were significantly higher than those for commercial Env which suggests that Env-CD4 proteins based on Asian-lineage strains may have more specific binding towards the Asian-American strain of ZIKV specific antibodies in Mexican patients. Alternatively, lower purity or stability of commercial Env may account for such differences. As the UK population is largely naive to flavivirus except those vaccinated against yellow fever virus and tick-borne encephalitis virus [36], the sero-diagnosis of ZIKV and the evaluation of immunogenicity during upcoming clinical trials in the UK is feasible. However, the difficulty in sero-diagnosis in flaviviral endemic areas, such as areas with dengue virus prevalence, makes the diagnosis more challenging due to antibody cross-reactivity [37–40], thus perhaps requiring ZIKV neutralisation assays to provide a correct sero-diagnosis which is still considered to be the “gold standard” [41–43]. To assess ZIKV antibody neutralisation activity in vitro and correlate the endpoint reciprocal antibody titers with the level of neutralising antibodies, a Zika plaque reduction and neutralisation test (PRNT) was performed which suggested that the reciprocal titers from the ELISA assay could be correlated with neutralising titers (ND50). This may be relevant during human clinical trials to monitor the levels of neutralising antibodies against ZIKV Env. It was previously shown in some studies that anti-dengue titers from the ELISA did not always correlate with neutralizing antibody titers unlike other flaviviruses, and this is yet to be determined for ZIKV [44]. 1 out of 10 control sera (BD4), had a detectable ND50 titer of 15.5. It is possible that this patient had been previously exposed to ZIKV or other flaviviruses or vaccinated against the yellow fever virus, but as these were blood donor samples, the information is not available. It would be of interest to further determine the cross-reactivity of the ZIKV Env in patients exposed to a different set of flavivirus (YFV, TBEV, JEV, DENV, etc.), with the main challenge of sourcing retrospective serum collection of both flavivirus vaccinated or flavivirus exposed individuals.

In conclusion, we have achieved large-scale production of an Asian-lineage ZIKV Env recombinant protein in mammalian cells, and by attaching a CD4 fusion tag at the end of ZIKV Env, we have increased the protein yield up to 10 times. The resulting recombinant proteins were recognised by anti-sera from ChAdOx1 ZIKV prME_ΔTM vaccinated mice and sera from patients with confirmed exposure to ZIKV. In addition, our ZIKV proteins are Asian-lineage based and presented high purity, whereas commercial ZIKV Env proteins and ELISA kits are based on an African strain. Besides further applications (basic research, diagnoses), our fused or single Env proteins might be useful to evaluate the humoral immune response to ZIKV in upcoming clinical trials of novel ZIKV vaccines with the added value of the correlation of neutralising capacity against ZIKV Env.

## Materials and methods

### Expression and purification of recombinant ZIKV envelope proteins (Env-CD4 and Env)

The pre-membrane (prM) and envelope (Env) gene sequence for Zika virus were synthesized from Geneart as a DNA fragment based on the sequence of an isolate from the 2013 French Polynesian ZIKV outbreak (GenBank: AHZ13508.1). The ZIKV synthetic gene was codon optimized for efficient expression in mammalian cells. The protein expression and purification was carried out at the Oxford Protein Production Facility (OPPF- UK), Harwell, UK. Briefly, in order to select constructs with best expression profiles in mammalian cells, constructs encoding different lengths of ZIKV prM-Env (amino acid residues 1–589, 1–595, 1–603 and 1–612) and ZIKV Env (amino acid residues 187–589, 187–595, 187–603 and 187–612) were amplified by PCR using *Phusion*™ *Flash High Fidelity* polymerase and cloned into pOPINTTGneo or pOPINTTGneo-3C-CD4 expression vectors via In-Fusion™ cloning and transformed into OmniMaxII T1-phage resistant cells (Invitrogen) in 96-tube format as previously described [45]. The plasmids were extracted using a QIAgen BioRobot 8000 with the Wizard SV96 minprep kit (Promega). The content of each plasmid was verified by PCR screening using pOPIN forward primer (a standard T7 forward primer) and a gene specific reverse primer and confirmed by DNA sequencing. For both constructs a 6x histidine tag was incorporated at the C-terminus. The expression screening was carried out in HEK293T cells according to the protocol by Nettleship et al. [24]. Briefly, 1 ml HEK 293 T cells were grown for 24 h in 24-well plates to reach ~ 70% confluency and transfected with each pOPIN construct using the GeneJuice™ transfection reagent (Novagen). To analyse the secretion of proteins into the media, the culture supernatant was harvested 3 days post transfection and analysed by SDS-PAGE using pre-cast Novex ® 4–12% gel in NuPAGE ® MOPS running buffer (Invitrogen). The His-tagged proteins were detected by Western blotting using 6x- His tag antibody (His.H8, Thermo Scientific). Based on the expression screen, the constructs encoding the Zika prM-Env (amino acid residues 1–589) in pOPINTTGneo and pOPINTTGneo-3C-CD4 were chosen for large scale protein expression using the Expi293™ Expression System Kit according to the manufacturer’s protocol. The supernatant containing the Zika prME was harvested after 96 h. Secreted protein was purified by automated immobilized metal affinity chromatography followed by gel filtration chromatography on ÄKTAxpress unit using the method of Nettleship et al. [24, 25]. Briefly, 200 mL of sample was loaded onto a 5 ml HisTrap FF column (GE Healthcare) before washing with 50 mL of 50 mM Tris, pH 7.5, 500 mM NaCl, 30 mM imidazole. Elution from the HisTrap FF column was performed with 50 mM Tris, pH 7.5, 500 mM NaCl, 500 mM imidazole and the eluted sample was injected directly onto a HiLoad 16/600 Superdex 200 column. Size exclusion chromatography was performed using 20 mM Tris pH 7.5, 200 mM NaCl. Resulting fractions were analysed by SDS-PAGE using InstantBlue protein stain (coomassie stain) and concentrated using an Amicon 400 concentrator with 30 kDa molecular weight cut-off membrane for subsequent immunological studies.

### Silver stain and Western blot

Silver staining of purified Zika virus envelope proteins (Env-CD4 and Env) from this study and commercially available recombinant Zika virus envelope protein expressed in insect cells (Aalto Bio reagents No. AZ6312) was carried out to assess purity according to the standard protocol using the Pierce Silver Stain Kit (No. 24612). Purified ZIKV envelope proteins were added to Laemmli sample buffer containing 20 mM β-mercaptoethanol. Samples were boiled for 3 mins and loaded on a Mini-PROTEAN TGX protein gel (reducing conditions) with a protein marker (Bio-Rad, Precision Plus Protein™ WesternC™ standards). For samples under non-reducing conditions, non-reducing sample buffer was used instead. For silver stain, PAGE gels were stained with Pierce Silver Stain Kit according to the manufacturer’s protocol. For Western blot, proteins were transferred onto nitrocellulose membranes (Bio-Rad Trans-Blot® TurboT^M^). Membranes were blocked with 5% skim milk in 0.1% PBS/T for 1 h and then incubated with one of the following antibodies; (1). 1:500 dilutions of a mouse anti-flavivirus group antigen monoclonal antibody (catalogue no. MAB10216, Millpore) (2). 1:500 dilutions of mice sera following a vaccination with ChAdOx1 ZIKV prME_ΔTM [30] (3). 1:1000 anti-Zika Env monoclonal antibody (mouse mAb to Zika Env protein, AZ1176, Aalto BioReagents) (4). 1:2000 anti-His antibody (mouse 6x-His Tag Antibody, MA1–21315, ThermoFisher Scientific). After washing with PBS/T, membranes were incubated for 1 h with a HRP-conjugated goat anti-mouse IgG (Bio-Rad Cat. 170–6516). Finally, membranes were washed again using PBS/T and incubated with a chemiluminescent substrate (Clarity™ Western ECL Blotting Substrates, BIO-RAD); signal was detected using a chemiluminescent Western blot imaging system (Image Lab, Bio-Rad).

### Immunisation of mice

Mice were vaccinated intramuscularly with a single dose of chimpanzee adenoviral vectored vaccine (ChAdOx1) encoding ZIKV prME_ΔTM or unrelated malarial antigen (cCSP) as control vaccines at dose of 1 × 10^8^ infectious units, as described [30].

### Mouse sera IgG ELISA

Anti-Zika envelope antibody concentrations were measured by a specific IgG enzyme-linked immunosorbent assay (ELISA) to recombinant Zika Env antigens. Briefly, Nunc Maxisorp Immuno ELISA plates were coated with Zika virus envelope antigens (commercial, Env-CD4 and Env) diluted in PBS to a final concentration of 2 μg/ml and left at RT overnight. Plates were washed 6 times with PBS/0.05% Tween (PBS/T) and blocked with 300 μl with Pierce™ protein-free (PBS) Blocking buffer (ThermoFisher) for 2 h at RT. Mice sera were obtained 3 months after single vaccination with ChAdOx1 ZIKV prME_ΔTM and unrelated control (cCSP) [30]. Mice sera reactive to ZIKV Env or reactive to an unrelated control antigen (cCSP) were added and serially diluted 3-fold down in PBS/T with 50 μl per well as final volume and incubated for 2 h at RT. Following washing 6 times with PBS/T, bound antibodies were detected following a 1 h incubation with 50 μl of alkaline phosphatase-conjugated antibodies specific for whole mouse IgG (Sigma, A3562-5ML). Following a further 6 washes with PBS/T, development was achieved using 100 μl of 4-nitrophenylphosphate diluted in diethanolamine buffer and the absorbance values at OD405 were measured and analysed using a CLARIOstar instrument (BMG Labtech). Serum antibody endpoint titers were defined by an absorbance value three standard deviations greater than the average OD405 of the control (cCSP) at 1:450 sera dilution.

### Human sera IgG ELISA

A total of sixteen sera from patients with RT-PCR confirmed Zika virus infections from Mexico were used for an IgG ELISA assay to measure the antibodies against recombinant Zika virus envelope antigens (Env-CD4). As controls, serum samples from ten healthy individuals from a non-endemic area were used, but no information on previous vaccination, travel to flavivirus endemic area or previous disease was available. Briefly, human sera were diluted in Nunc Maxisorp Immuno ELISA plates coated with Zika virus envelope antigens (Env-CD4 or commercial Env) in PBS to a final concentration of 5 μg/ml. Following blocking, the sera was incubated for 1 h, then plates were washed 6 times with PBS/T (0.05%), bound antibodies were detected by using a goat Anti-Human IgG-alkaline phosphatase-conjugated antibody (Sigma, A3187-5ML). Development was performed using 4-nitrophenylphosphate diluted in diethanolamine buffer and absorbance values at OD405 were measured on a CLARIOstar instrument (BMG Labtech). Serum antibody endpoint titers were defined by absorbance value three standard deviations greater than the average OD405 of control sera pool at 1:300 sera dilution.

### Zika plaque reduction and neutralisation assay

Sixteen sera from Mexican patients and ten sera from healthy donors were tested in duplicate by a Zika plaque reduction and neutralisation test (PRNT). 60 pfu of the ZIKV Asian strain (PRVABC59) were mixed with doubling dilutions of human sera ranging from 1:10 to 1:160 and incubated at 37 °C for 1 h to allow the serum to neutralise the virus. This mixture was then added to a confluent monolayer of Vero cells that had been seeded onto a 12 well plate at a density of 2 × 10^5^ cells per well. After a 1.5 h incubation at room temperature, the viral inoculum was removed and cells were overlaid with 1 ml of a 1% agarose solution containing 2% HI-FCS. The plates were then incubated for 4 days at 37 °C, 5% CO2, 95% humidity. Cells were fixed and stained with 1 ml of toluidine blue solution (0.1% toluidine blue, 2.7% formaldehyde, 1x PBS) and incubated at RT overnight to allow the stain to soak through the agarose. The plaque number per treatment was compared to the plaque number in the control samples and neutralising antibody titers were expressed as the serum dilution yielding a 50% plaque number reduction (ND50). ND50 titers were calculated for each serum using the Spearman-Karber formula. The cut off titer for positivity was set as 10.

### Data analysis and statistics

All data analysis and statistics were performed using prism 7 software (GraphPad, Software, US). *P* values and R^2^ values reflect Pearson correlation tests for determining the correlation between the endpoint reciprocal titer values from ELISA assay and ND50 titers from PRNT assay. *P* value (< 0.0001) between Env-CD4 and commercial Env was determined by pairwise t-tests.

## Additional files

Additional file 1: Structural alignment of the ZIK Env sequence of an isolate from the 2013 French Polynesian Zika outbreak with that of Dengue virus (Protein Data Bank ID: 1OAN) [31]. (PDF 113 kb)

Additional file 2: Summary of small scale HEK secretions by WB using a His tag antibody. Summary of small scale HEK secretions by WB using a His tag antibody from all 16 plasmid constructs encoding prM-Env and Env in pOPINTTGneo or pOPINTTGneo-3C-CD4 expression vectors. Green colour indicates good expression with 3 consistent results whereas yellow colour shows weak expression (2 consistent results) and red colour means no expression (1 or 0). (PDF 33 kb)

Additional file 3: PAGE-Silver stain of ZIKV Env-CD4 proteins under reducing and non-reducing conditions. Two concentrations of Asian-lineage ZIKV Env-CD4 (500 ng and 250 ng) were used. There is lesser degree of CD4 fusion tag cleavage into Env and CD4 at non-reducing conditions. (PDF 39 kb)
