# Supplementary figures and images for: Optimization of Zika virus envelope protein production for ELISA and correlation of antibody titers with virus neutralization in Mexican patients from an arbovirus endemic region
Source: Virol J. 2018 Dec 27;15:193. doi: 10.1186/s12985-018-1104-6 (PMC6307127; doi:10.1186/s12985-018-1104-6)

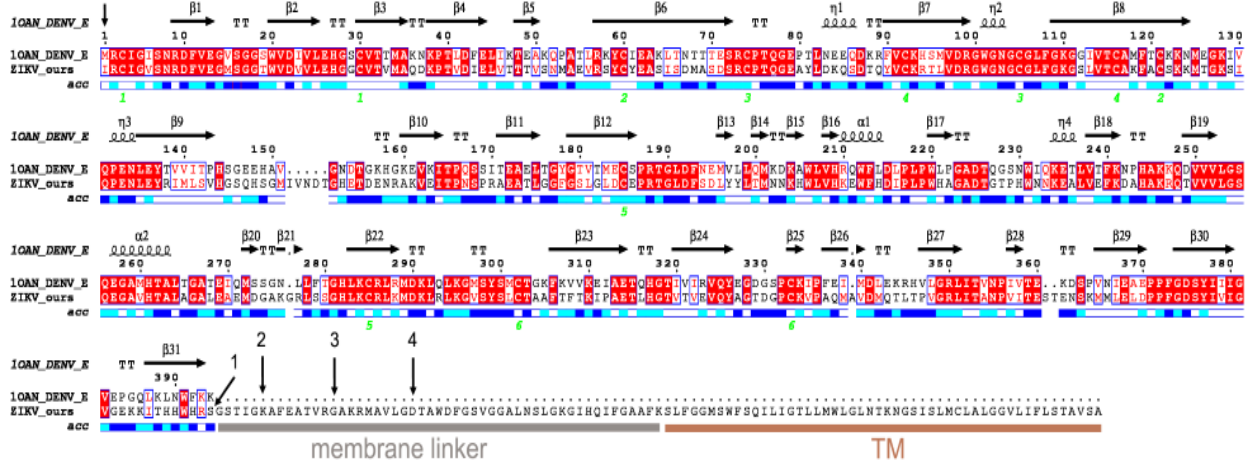

Supplement: Supplementary file 1 — Structural alignment of the ZIK Env sequence of an isolate from the 2013 French Polynesian Zika outbreak with that of Dengue virus (Protein Data Bank ID: 1OAN) [31]. (PDF 113 kb) [file 12985_2018_1104_MOESM1_ESM.pdf]

# Asian ZIKV Env-CD4 (ng)

Reducing

Non-Reducing

500

250

500

250

kDa

100

75

50

37

25

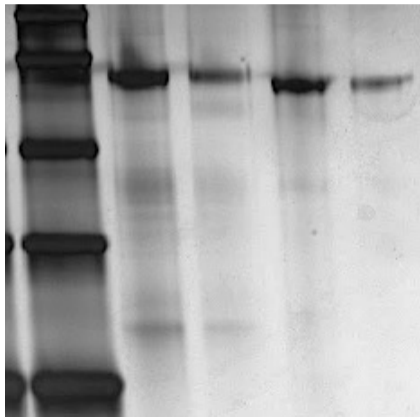

← Env-CD4

← Env

← CD4

Supplement: Supplementary file 3 — PAGE-Silver stain of ZIKV Env-CD4 proteins under reducing and non-reducing conditions. Two concentrations of Asian-lineage ZIKV Env-CD4 (500 ng and 250 ng) were used. There is lesser degree of CD4 fusion tag cleavage into Env and CD4 at non-reducing conditions. (PDF 39 kb) [file 12985_2018_1104_MOESM3_ESM.pdf]
