# Supplementary material for: Optimization of Zika virus envelope protein production for ELISA and correlation of antibody titers with virus neutralization in Mexican patients from an arbovirus endemic region
Source: Virol J. 2018 Dec 27;15:193. doi: 10.1186/s12985-018-1104-6 (PMC6307127; doi:10.1186/s12985-018-1104-6)
Supplement: Supplementary file 2 — Summary of small scale HEK secretions by WB using a His tag antibody. Summary of small scale HEK secretions by WB using a His tag antibody from all 16 plasmid constructs encoding prM-Env and Env in pOPINTTGneo or pOPINTTGneo-3C-CD4 expression vectors. Green colour indicates good expression with 3 consistent results whereas yellow colour shows weak expression (2 consistent results) and red colour means no expression (1 or 0). (PDF 33 kb) [file 12985_2018_1104_MOESM2_ESM.pdf]

Description (a.a)	Vector	CD4	Secretions (N=3)
prM-Env 1-612	pOPINTTGneo	(-) CD4	
prM-Env 1-603	pOPINTTGneo		
prM-Env 1-595	pOPINTTGneo		
prM-Env 1-589	pOPINTTGneo		
prM-Env 1-612	pOPINTTGneo -3C -CD4	(+) CD4	
prM-Env 1-603	pOPINTTGneo -3C -CD4		
prM-Env 1-595	pOPINTTGneo -3C -CD4		
prM-Env 1-589	pOPINTTGneo -3C -CD4		
Env 187-612	pOPINTTGneo	(-) CD4	
Env 187-603	pOPINTTGneo		
Env 187-595	pOPINTTGneo		
Env 187-589	pOPINTTGneo		
Env 187-612	pOPINTTGneo -3C -CD4	+CD4	
Env 187-603	pOPINTTGneo -3C -CD4		
Env 187-595	pOPINTTGneo -3C -CD4		
Env 187-589	pOPINTTGneo -3C -CD4		
